# Lidocaine relaxation in isolated rat aortic rings is enhanced by endothelial removal: possible role of K_v_, K_ATP_ channels and A_2a_ receptor crosstalk

**DOI:** 10.1186/s12871-016-0286-y

**Published:** 2016-12-03

**Authors:** Aryadi Arsyad, Geoffrey P. Dobson

**Affiliations:** 1 Heart, Trauma and Sepsis Research Laboratory, Australian Institute of Tropical Health and Medicine, College of Medicine and Dentistry, James Cook University, 1 James Cook Drive, 4811 Queensland, Australia; 2 Physiology Department, Medical Faculty, Hasanuddin University, Jl. Perintis Kemerdekaan, Km. 10, 90213 Tamalanrea, Makassar Indonesia

**Keywords:** Rat aorta, Lidocaine, Relaxation, Vasodilation, Endothelium, Nitric oxide, Redox stress

## Abstract

**Background:**

Lidocaine is an approved local anesthetic and Class 1B antiarrhythmic with a number of ancillary properties. Our aim was to investigate lidocaine’s vasoreactivity properties in intact versus denuded rat thoracic aortic rings, and the effect of inhibitors of nitric oxide (NO), prostenoids, voltage-dependent K_v_ and K_ATP_ channels, membrane Na^+^/K^+^ pump, and A_2a_ and A_2b_ receptors.

**Methods:**

Aortic rings were harvested from adult male Sprague Dawley rats and equilibrated in an organ bath containing oxygenated, modified Krebs-Henseleit solution, pH 7.4, 37 °C. The rings were pre-contracted sub-maximally with 0.3 μM norepinephrine (NE), and the effect of increasing lidocaine concentrations was examined. Rings were tested for viability after each experiment with maximally dilating 100 μM papaverine. The drugs 4-aminopyridine (4-AP), glibenclamide, 5-hydroxydecanoate, ouabain, 8-(3-chlorostyryl) caffeine and PSB-0788 were examined.

**Results:**

All drugs tested had no significant effect on basal tension. Lidocaine relaxation in intact rings was biphasic between 1 and 10 μM (Phase 1) and 10 and 1000 μM (Phase 2). Mechanical removal of the endothelium resulted in further relaxation, and at lower concentrations ring sensitivity (% relaxation per μM lidocaine) significantly increased 3.5 times compared to intact rings. The relaxing factor(s) responsible for enhancing ring relaxation did not appear to be NO- or prostacyclin-dependent, as L-NAME and indomethacin had little or no effect on intact ring relaxation. In denuded rings, lidocaine relaxation was completely abolished by K_v_ channel inhibition and significantly reduced by antagonists of the MitoK_ATP_ channel, and to a lesser extent the SarcK_ATP_ channel. Curiously, A_2a_ subtype receptor antagonism significantly inhibited lidocaine relaxation above 100 μM, but not the A_2b_ receptor.

**Conclusions:**

We show that lidocaine relaxation in rat thoracic aorta was biphasic and significantly enhanced by endothelial removal, which did not appear to be NO or prostacyclin dependent. The unknown factor(s) responsible for enhanced relaxation was significantly reduced by K_v_ inhibition, 5-HD inhibition, and A_2a_ subtype inhibition indicating a potential role for crosstalk in lidocaine’s vasoreactivity.

## Background

Lidocaine is a local amide-type cationic anesthetic, which acts by blocking voltage-dependent Na^+^ fast channels in excitable cells (EC50, 50–100 uM) [1]. At lower concentrations, lidocaine is an approved Class 1B antiarrhythmic [2] and exerts anti-inflammatory [3], neuroprotective [4], energy-lowering [5], anti-ischemic [6, 7], anti-oxidant [8, 9] and platelet-neutrophil interactive [10, 11] properties.

Lidocaine has also been shown to exert a number of vasomodulatory properties in isolated vessels including: 1) endothelium-independent relaxation [12, 13], and 2) vascular smooth muscle relaxation [12, 14, 15] or contraction [15–19] properties. The apparent paradoxical nature of lidocaine on vascular smooth muscle is often explained as being dose-dependent with vasoconstriction of peripheral blood vessels occurring at low concentrations of lidocaine (~5 uM) and vasodilation at higher levels (>50 uM) [14, 16, 20, 21]. In the rat carotid artery, Kinoshita further proposed that lidocaine may impair the vasodilator response via the activation of ATP-sensitive K^+^ channels which may exacerbated by hypoxia [19]. Earlier the same group showed that in pre-contracted denuded rat aortic rings that acidification promoted lidocaine relaxation and alkanization led to vasoconstriction [18].

These confounding effects of lidocaine vasoreactivity appear to be linked to differential modulation of multiple channels including Na^+^ channels [2], inwardly-rectifying K^+^ channels [22], Ca^2+^ channels [13, 23] and/or K_ATP_ channels [18, 19]. Vasodilation may involve nitric oxide (NO) [24–26], redox regulation [9] and possible convergence of a multitude of downstream cAMP and cGMP signalling cascades that lead to changes in cytosolic Ca^2+^ [27]. Hollmann and colleagues, for example, identified lidocaine and G-protein coupled receptor systems as potential intracellular signalling mechanisms, and the Gq protein subunit as a possible common target [28]. In 2003, Benkwitz et al., also showed that the G_i_ protein subunit was enhanced by lidocaine, and that it was potentiated adenosine A1-receptor signalling [29]. The group proposed that lidocaine was not an A1-receptor agonist but enhanced adenosine-A1 receptor signalling separate from its local anesthetic Na^+^ channel properties [29]. The aim of the present study was to investigate the nature of lidocaine relaxation in isolated rat thoracic aortic rings, and examine the effect of inhibitors of NO, prostenoids, K_v_ channels, K_ATP_ channels, and adenosine A_2a_ and A_2b_ receptors. Adenosine A_2_ receptors were chosen because they are widely known to modify vascular tone [30], and may therefore be involved in possible crosstalk in lidocaine relaxation [29].

## Methods

### Animals

Male Sprague Dawley rats (300–350 g, *n* = 72) were fed ad libitum and housed in a 12-h light/dark cycle. On the day of the experiment rats were anaesthetised with Na-thiopentone (100 mg/kg). Animals were treated in accordance with the Guide for the Care and Use of Laboratory Animals published by the US National Institutes of Health (NIH Publication No. 85-23, revised 1996). The James Cook University ethics approval number for the studies was A1535. Lidocaine hydrochloride was sourced as a 2% solution (Ilium) from the local Pharmaceutical Suppliers (Lyppard, Queensland). All other chemicals, drugs and inhibitors were purchased from Sigma Aldrich (Castle Hill, NSW).

### Aortic ring preparation and organ bath tension measurements

The thoracic cavity of anesthetized rats was opened and the thoracic aorta was harvested and placed in a modified ice-cold solution of Krebs Henseleit (118 mM NaCl, 4.7 mM KCl, 1.2 mM Na_2_PO_4_, 0.5 mM MgCl_2_, 1.12 mM CaCl_2_, 25 mM NaHCO_3_, 0.03 mM EDTA) pH 7.4 with 11 mM glucose. The aorta was carefully dissected from surrounding fat and connective tissue and cut into short transverse segments. Intact aortic rings were isolated from each rat and used without further processing. In those studies that required removal of the endothelium, intact rings were denuded by gently rubbing the intimal surface of the vessel segment with a smooth metal probe. Successful removal of the endothelium was assessed by testing the aortic ring for a vasodilatory response to 10 μM acetylcholine (final concentration).

**Fig. 1:**
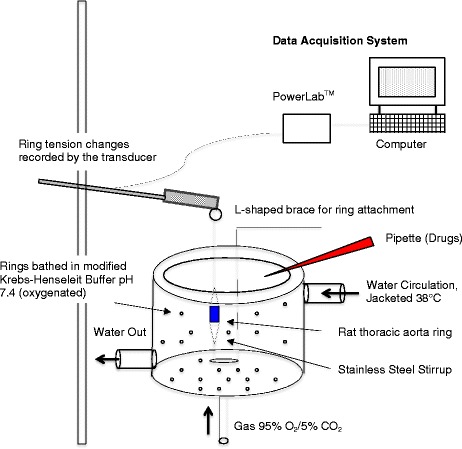
A graphical scheme of the rat thoracic aorta ring apparatus for isometric force measurements, receptor sensitivity and smooth muscle-endothelial interactions. See Methods for details

After preparation, intact or denuded aortic rings (3 to 4 mm long) were equilibrated in a standard 10 ml volume organ bath (Radnotti Glass, ADinstruments, NSW, AUS) containing modified Krebs Henseleit (see above) and continuously bubbled with 95% O_2_ and 5% CO_2_ at 37 °C for 15 min (zero tension) (Fig. 1). The rings were vertically mounted on small stainless steel triangles, stirrups and connected to an isometric force transducer (PANLAB, distributed by ADInstruments as MLT 0201/RAD, NSW, AUS) coupled to a computer based data acquisition system (PowerLab, ADInstruments) and data recording software LabChart 7 (ADInstruments Pty Ltd., Castle Hill, Australia) (Fig. 1).

The ring tension was manually adjusted to 1.5 g and equilibrated for 60 min. A tension of 1.5 g was chosen from the literature for thoracic aortic rings prepared from 300 to 400 g rats [18, 31] and preliminary studies verified this tension. During equilibration, the solution was changed in 15 min intervals. The aortic rings were then washed with freshly prepared Krebs Henseleit buffer pH 7.4 and the tension was readjusted to 1.5 g tension. Each preparation was sub-maximally contracted using 3 μl of 0.1 mM NE (0.3 μM final concentration) [15, 32]. Those aortic rings that failed to contract were discarded. Ten microliters of 10 mM acetylcholine (10 μM final concentration) was applied to confirm the presence or absence of an intact endothelium in all preparations. Acetylcholine will induce rapid relaxation of precontracted rings if the endothelium is intact and if the endothelium is removed (or denuded) the rings will remain in contracted state [33]. Aortic rings were considered intact if the relaxation induced by 10 μM ACh was greater than 80%, and the aortic ring was assumed denuded if relaxation was less than 10%.

Rings were contracted at least two more times before each experiment until a reproducible contractile response was obtained. Ten to 15 min after this state was achieved the experiment was commenced because preliminary studies showed that the increase in tension and plateau from 0.3 μM of NE was reached at 10 min and remained at this plateau level for over 60 min, the time course of each experiment.

### Lidocaine relaxation in intact and denuded rat aortic rings: a scheme of the experimental protocol

Lidocaine-HCl was added into the oxygenated organ bath containing KH solution to obtain 1, 5, 10, 50, 100, 500 and 1000 μM lidocaine concentrations. The change in tension of pre-contracted intact or denuded rings was measured. Responsiveness was defined as % relaxation per μM lidocaine. The inhibitors used in this study were incubated in organ bath 20–30 min before NE was administered followed by lidocaine incremental administration. These included 1). 100 μM N^G^-nitro-L-arginine Methyl Ester (L-NAME) (nitric oxide synthetase inhibitor) and 10 μM indomethacin (cyclooxygenase or prostaglandin inhibitor e.g. prostacyclin). NO and prostacyclin are two major endothelial derived relaxation factors (EDRF), and the inhibitors were only applied in endothelium intact aortic rings. 2). 1 mM 4-aminopyridine (4-AP) (Non-selective voltage-dependent *K* ^+^-channel blocker of the Kv1 to Kv4 families rather than Kv7 channels) [34–36], 10 μM glibenclamide (Non-selective SarcK_ATP_ channel blocker) [37, 38] and 1 mM 5-hydroxydecanoate (5-HD) (Non-selective MitoK_ATP_ channel blocker) [39], and Na^+^/K^+^-ATPase inhibitor (100 μM ouabain) [40]. While 5-HD is commonly used in the literature as a specific MitoK_ATP_ channel blocker [41], Hanley and colleagues have shown that 5-HD is not a selective inhibitor of mitochondrial K_ATP_ channels but can act a substrate for the mitochondrial outer membrane enzyme acyl-CoA synthetase in the beta-oxidation pathway [42]), and it is also capable of playing a role as an inhibitor of sarcolemmal K_ATP_ channels in the presence of ATP (which was not the case in our study) [43]. These inhibitors were applied to intact endothelium rings in the presence of L-NAME and indomethacin, and without the presence of L-NAME and indomethacin in denuded aortic rings, and 3) The adenosine A_2a_ receptor inhibitor, 100 μM 8-(3-chlorostyryl) caffeine (CSC) [44–46], and the A_2b_ receptor inhibitor, 10 μM 8-(4-(4-(4-chlorobenzyl)piperazine-1-sulfonyl)phenyl)-1-propylxanthine (PSB-0788) [47]. These high affinity antagonists have been used in rodent studies with reported K_i_ values of 24 nM for CSC [48] and 0.393 nM for PSB-0788 [47]. CSC has also been shown to be 520-fold selective for A2a-adenosine receptors in radioligand binding assays in the rat brain (K_i_, 54 nM) with little or no effect on A1 receptors [44]. The inhibitors were applied to isolated rat aortic rings in an oxygenated medium. At the end of each experiment, the rings were tested for viability (or patency) by being maximally dilated with 100 μM papaverine, and relaxation was expressed as percentage of maximal relaxation to papaverine [40, 49].

### Statistics

Values are expressed as mean ± SEM. The number of rats was selected from a priori G-power analysis to achieve a level of 1.0. Values are expressed as mean ± SEM. All data was tested for normality using *Kolmogorov-Smirnov* test. Relaxation responses to lidocaine were analysed for homogeneity of variances followed by two-way ANOVA coupled with the *Bonferroni* post-hoc test for individual data point comparisons. The alpha level of significance for all experiments was set at *p* < 0.05.

## Results

### Effect of increasing lidocaine on relaxation in intact and denuded rings

#### Intact rings

**Fig. 2:**
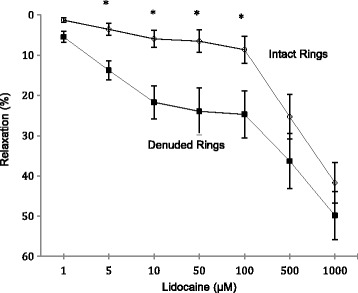
Concentration response curves to lidocaine in intact and denuded isolated rat aortic rings. Relaxation is expressed as percent of maximal relaxation to 100 μM papaverine. Points represent mean ± S.E.M of aortic rings. *P < 0.05 statistical difference in responses between the intact and denuded rings. Lidocaine concentrations are on a log scale. Total animals *n* = 12

The gram tension produced with NE administration in endothelium intact rings was not significantly different from denuded aortic rings. Lidocaine produced a concentration-dependent, biphasic relaxation relationship in intact and denuded rat aortic rings (Fig. 2). The percentage relaxation in intact rat aortic rings was 1.3, 6.0, 8.6 and 41.7% at 1, 10, 100 and 1000 uM lidocaine respectively. The first relaxation phase was between 1 and 10 uM (Phase 1) and the second phase was from 10 to 1000 uM lidocaine (Phase 2) (log concentration scale) (Fig. 2). The percentage relaxation per μM lidocaine (ring responsiveness) was 0.47% from 1 to 10 μM, 0.029% between 10 and 100 μM and 0.037% increase per μM between 100 and 1000 μM lidocaine. The maximum relaxation from 1 to 1000 μM lidocaine in intact rings was 40.4%.

#### Denuded rings

Removal of the endothelium significantly increased Phase 1 relaxation responsiveness from 0.47 to 1.80% per μM lidocaine between 1 and 10 μM (Fig. 2). Interestingly, above 10 μM removing the endothelium had little or no significant effect on ring responsiveness to increasing lidocaine compared to intact rings. From 10 to 100 μM, % relaxation per μM was 0.033% and from 100 to 1000 μM was 0.028% (Fig. 2). However, despite this similar responsiveness, at each lidocaine concentration up to 100 μM, the absolute percentage relaxation was significantly higher in denuded rings than intact rings. The absolute % relaxation in denuded rings was 5.5, 14, 22, 24, 25, 36 and 50% at 1, 5, 10, 50, 100, 500 and 1000 uM lidocaine respectively (Fig. 2). Thus the effect of removing the endothelium was to significantly enhance ring *sensitivity* or *responsiveness* at lower lidocaine concentrations (1 to 10 μM) but not in the higher range (10 to 1000 μM), even though absolute relaxation values were significantly higher at each lidocaine concentration (1 to 1000 μM) in denuded versus intact rings (Fig. 2).

### Effect of L-NAME and indomethacin in intact aortic rings

**Fig. 3:**
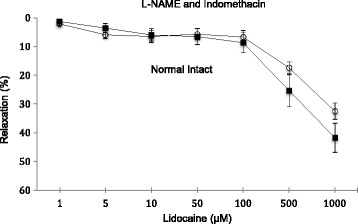
Concentration-response curves to lidocaine with and without the presence of L-NAME + indomethacin in intact isolated rat aortic rings. Relaxation is expressed as percent of maximal relaxation to 100 μM papaverine. Points represent mean ± S.E.M of aortic rings. *P < 0.05 statistically different in the presence of L-NAME + indomethacin compared to control on intact rings. Lidocaine concentrations are on a log scale. Total animals *n* = 12

In intact aortic rings, pre-treatment with L-NAME and indomethacin did not significantly change lidocaine relaxation from 1 to 1000 μM, although there was a trend towards inhibition at higher concentrations (Fig. 3). Between 1 and 10 μM, the change in relaxation was 0.44% per μM, 0002% per μM between 10 and 100 μM and 0.029% per μM from 100 to 1000 μM. At 500 μM lidocaine, the % relaxation was 17% (32% lower than intact rings) and at 1000 μM lidocaine was 32% (24% lower than intact rings), but the differences were not significant (Fig. 3).

### Effect of K_v_, SarcK_ATP_, MitoK_ATP_ and Na^+^/K^+^ ATPase antagonists on relaxation in intact and denuded rings

**Fig. 4:**
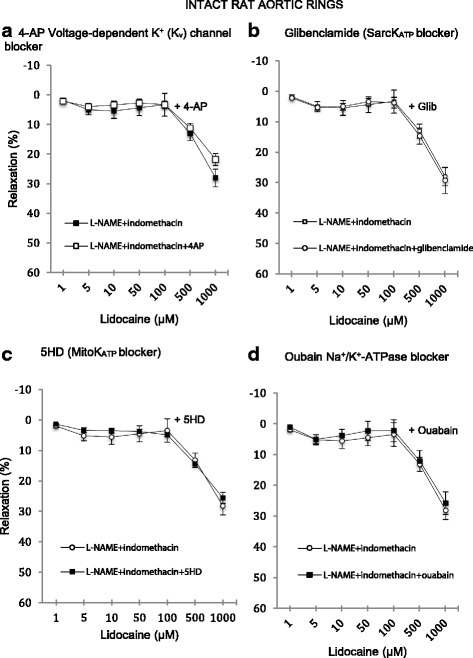
Concentration-response curves to lidocaine with and without the presence of specific ion channel blockers in intact isolated rat aortic rings. **a** In the presence of 1 mM 4-aminopyridine. **b** In the presence of 1 mM 5-Hydroxydecanoate. **c** In the presence of 10 μM glibenclamide. **d** In the presence of 100 μM ouabain. Relaxation is expressed as percent of maximal relaxation to 100 μM papaverine. Points represent mean ± S.E.M of aortic rings in the presence of L-NAME and indomethacin. *P < 0.05 statistical difference in responses between the presence and the absence of inhibitors on intact rings. Lidocaine concentrations are on a log scale. Total animals *n* = 16

The effects of voltage-dependent K_v_, SarcK_ATP_, mitoK_ATP_ and Na^+^/K^+^-ATPase antagonists on lidocaine relaxation in intact rat aortic rings are shown in Fig. 4. After pre-contracted with NE, ring basal tensions were 3.3 ± 0.09 g; 3.5 ± 0.17 g; 3.4 ± 0.09 g; 3.4 ± 0.14 g (*n* = 8 each) for 4-AP, glibenclamide, 5-HD and ouabain groups, respectively; and not significantly different from NE with L-NAME and indomethacin controls (3.2 ± 0.19 g, *n* = 8). In endothelial intact aortic rings, exposure of rings to these antagonists did not alter lidocaine-induced relaxation compared to controls (Fig. 4).

**Fig. 5:**
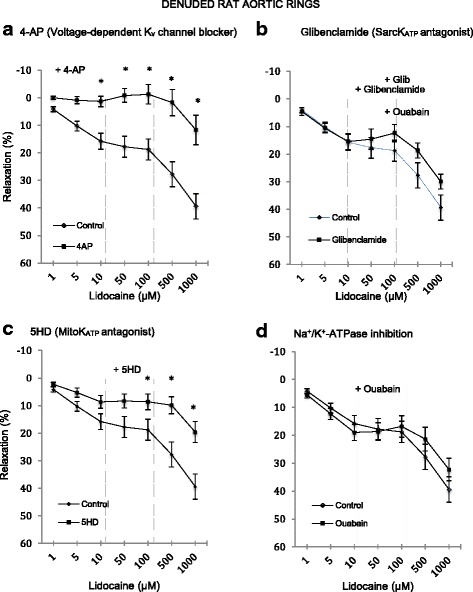
Concentration-response curves to lidocaine with and without the presence of specific ion channel blockers in denuded isolated rat aortic rings. **a** In the presence of 1 mM 4-aminopyridine. **b** In the presence of 1 mM 5-Hydroxydecanoate. **c** In the presence of 10 μM glibenclamide. **d** In the presence of 100 μM ouabain. Relaxation is expressed as percent of maximal relaxation to 100 μM papaverine. Points represent mean ± S.E.M of aortic rings in the presence of L-NAME and indomethacin. *P < 0.05 statistical difference in responses between the presence and the absence of inhibitors on intact rings. Lidocaine concentrations are on a log scale. Total animals *n* = 16

In denuded rings, the effect of 1 mM 4-AP was to totally abolish relaxation up to 500 μM after which relaxation was 12 ± 5% (*n* = 8) compared to 39 ± 5% in denuded controls (i.e. 4-AP led to a 70% decrease in relaxtion) (Fig. 5a). 4-AP inhibition was significant from 1 to 1000 μM lidocaine (*p* < 0.0001). The effect of glibenclamide (10 μM) had little or no effect on relaxation up to 10 μM lidocaine compared to denuded controls (Fig. 5b) and was ~20% lower at higher lidocaine concentrations; however, the differences were not significant. Exposure of denuded rings to 1 mM 5-HD led to ~50% decrease in lidocaine relaxation at 5 to 1000 μM lidocaine which was significant >50 μM (Fig. 5c). The presence of 100 μM ouabain, a Na^+^/K^+^-ATPase channel inhibitor, had little or no significant effect on lidocaine-induced relaxation (Fig. 5d).

### Effect of A_2a_ and A_2b_ antagonists in intact and denuded rat aortic rings

**Fig. 6:**
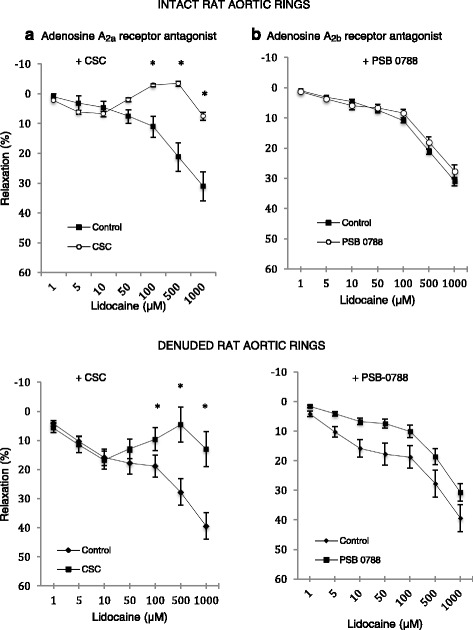
Concentration-response curves to lidocaine with and without the presence of adenosine A_2ab_ receptor blockers in intact and denuded isolated rat aortic rings. **a** (Adenosine A2a receptor antagonist) In the presence of 100 μM 8-(3-Chlorostyryl) caffeine. **b** (Adenosine A2b receptor antagonist) In the presence of 10 μM PSB-0788. Relaxation is expressed as percent of maximal relaxation to 100 μM papaverine. Points represent mean ± S.E.M of aortic rings in the presence of L-NAME and indomethacin. *P < 0.05 statistical difference in responses between the presence and the absence of inhibitors on intact rings. Lidocaine concentrations are on a log scale. Total animals *n* = 16

The basal tension of NE-precontracted CSC group was 3.1 ± 0.16 g and PSB-0788 groups 3.7 ± 0.07 g (*n* = 8 each) and not significantly different from controls (3.2 ± 0.19 g, *n* = 8). Adenosine A_2a_ antagonist 8-(3-chlorostyryl) caffeine (CSC) significantly decreased lidocaine relaxation in the intact rat aorta at 100 to 1000 μM (Fig. 6). Divergence began to occur at 50 μM lidocaine with relaxation values of 2, −2.8, −3.4 and 7.6% at 50, 100, 500 and 1000 μM lidocaine respectively. In direct contrast, the incubation with PSB-0788, an adenosine A_2b_ antagonist, did not modify lidocaine-induced relaxation curve at any concentration used in NE pre-contracted aortic rings (Fig. 6).

In denuded rings, the basal tension of aortic rings with the presence of CSC (2.5 ± 0.15 g) or PSB-0788 (3.1 ± 0.18 g) was not significantly different. CSC had no effect on relaxation up to 10 uM lidocaine then strongly inhibited relaxation up to 500 μM (Fig. 6). The maximum lidocaine relaxation was 13 ± 6%, which was significantly lower than control denuded rings (39 ± 5%, *p* < 0.0001) (Fig. 6). The adenosine A_2b_ receptor blocker PSB-0788 (10 μM) also decreased lidocaine relaxation by up to 50% but this effect was not significant (Fig. 6). At 10, 100, and 1000 μM lidocaine, the relaxation percentages were 7 ± 1, 10 ± 2, and 31 ± 3%, respectively compared to 16 ± 3, 19 ± 4, and 39 ± 5% in denuded controls (Fig. 6).

## Discussion

Despite decades of investigation, the mechanisms of lidocaine relaxation in the rat thoracic aorta, and muscular resistance arterioles are not fully understood [12, 14–16, 20, 21]. We report in isolated rat thoracic rings, pre-contracted with NE, that lidocaine relaxation was: 1) biphasic from 1 to 10 μM and 10 to 1000 μM, 2) significantly enhanced by endothelial removal, particularly from 1 to 100 μM, 3) not significantly affected in the presence of L-NAME- and indomethacin in intact rings, 4) abolished by 4-AP in denuded rings and significantly reduced by 5-HD, and to a lesser extent glibenclamide, and 5) significantly reduced by A_2a_ subtype antagonist from 100 to 1000 μM, but not A_2b_. We discuss the possible physiological significance of the biphasic nature of lidocaine relaxation, enhancement after endothelial removal, and potential role for crosstalk with the A_2a_ subtype and voltage-dependent K_v_ and K_ATP_ channels.

### Lidocaine relaxation was biphasic and endothelial dependent

We found in oxygenated, glucose-containing Krebs Henseleit buffer, pH 7.4 at 37 °C: 1) little or no change in relaxation in rat aortic rings at low lidocaine concentrations, and 2) a strong endothelial dependence which enhanced relaxation after its removal (Fig. 2). The data suggest that the presence of an intact endothelium acted like a “brake” to reduce lidocaine relaxation, and upon its removal activated some putative factor to enhance relaxation. Our findings are in contrast with those reported in rat cremaster skeletal muscle [14], epicardial porcine coronary arteries [50], human radial arterial rings [51], human mammary arteries [52], and rabbit carotid arteries [15, 53], where lidocaine at low levels potentiated vasoconstriction, and at high concentrations led to relaxation. Jembeck and Samuelson further reported in isolated rings from radial arteries that lidocaine led to significantly stronger contractions after the endothelium was mechanically removed [51]. Reasons for the differences are not clear at present but may relate to species, age, mode of sacrifice, physiological state, pre-contractile conditions activating different channels and receptors (e.g. alteration of the membrane smooth muscle potential with high K^+^ versus NE or phenylephrine to pre-contract isolated rings), tissue preparation, different endothelial removal procedures and possible damage, buffer conditions, temperature, P_O2_ availability, and the sequence of drug additions and concentrations. Another important difference is vessel type; we studied the rat thoracic aorta, which is a large, highly elastic artery that normally offers little resistance to flow but assists in coupling the heart, as a pump and pressure-generator, to the arterial system by changing aortic compliance not resistance [54].

That lidocaine relaxation occurred from 1 to 1000 uM is consistent with the study of Shan and colleagues who showed that lidocaine relaxed phenylephrine or KCl (60 mM) precontracted rat aortic rings in a concentration-dependent manner [13]. However, their study differed from ours because they showed lidocaine relaxation was not significantly modified by endothelium removal, and their aortic rings were obtained from rats sacrificed by stunning and cervical dislocation, not anesthesia [13]. Our study also agreed with Turan and colleagues who showed lidocaine relaxed phenylephrine-precontracted rabbit thoracic aorta intact and denuded rings, however, when lidocaine (1 to 100 μM) was applied 15 min before the addition of phenylephrine it produced contractions at high concentrations (up to 10 mM), and endothelium removal did not significantly affect contractile activity. This example demonstrates the dynamics of the pre-contractile state and the importance of specifying the sequence of drug administration, which can produce different results. Further studies are required to investigate these discrepancies in the thoracic aorta of rat and other species prepared from different modes of sacrifice, different precontracted states and basal tone.

### Lidocaine relaxation enhancement involves an endothelium-smooth muscle coupling and possible activation of K_v_ and K_ATP_ channels

Since lidocaine relaxation displayed a strong endothelial-dependence (Fig. 2), it suggested a possible role for NO release or activation of the cyclo-oxygenase pathway and/or their interactions with the adrenoreceptors on vascular smooth muscle. Surprisingly, we found little or no effect of either L-NAME and indomethacin on lidocaine relaxation (Fig. 3) indicating that the putative relaxing factor after endothelial removal was neither NO nor prostacyclin. Other unknown factor(s) must be released upon endothelial removal to enhance lidocaine relaxation. Another possibility is endothelial-dependent activation of smooth muscle voltage-dependent Kv channels and/or smooth muscle mitochondrial K_ATP_ channels, since we showed that 4-AP completely abolished relaxation (Figs. 3a and 4a) and 5-HD led to ~50% inhibition in denuded rings (Figs. 4c and 5c).

Enhanced lidocaine relaxation may also have come from changing the cellular redox state and reactive oxygen species (ROS) derived from NAD(P)H oxidases [55, 56], as it has been reported that lidocaine at higher concentrations protects against ROS attack in rabbit abdominal aorta [9]. Rogers and colleagues further showed that 4-AP-sensitive K_v_ channels are redox sensitive and contribute to H_2_O_2_-induced coronary vasodilation [57]. In summary, we conclude that enhanced lidocaine relaxation after endothelial removal does not appear to involve the direct activation of NO or prostanoid-linked pathways, and that other relaxing factors and downstream signalling pathways, possibly involving Kv and/or 5-HD sensitive K_ATP_ channels, are involved.

### Smooth muscle adenosine A_2a_ modulation may also be involved in the enhanced lidocaine relaxation

The present study also suggests an intriguing possibility for enhancing lidocaine relaxation may be activation of the A_2a_ receptor on vascular smooth muscle. A surprising result was that lidocaine relaxation above 50 μM in intact and denuded rat aortic rings was significantly inhibited by 75 to 100% in the presence of A_2a_ blocker 8-(3-chlorostyryl) caffeine (CSC) (Fig. 5). This implies that the A_2a_ receptor may be involved in the presence or absence of an intact endothelium. Assuming CSC has high specificity for A_2a_ receptors [44], this antagonist may reduce lidocaine relaxation from one or more of the following mechanisms: 1) Directly or indirectly increasing Ca^2+^ influx from extracellular sources such as L-type Ca^2+^ channels [58], 2) increasing the release of Ca^2+^ from intracellular stores (e.g. sarcoplasmic or endoplasmic) to increase cytosolic free Ca^2+^, and/or 3) increasing myofibrillar contractile sensitivity to existing free Ca^2+^ (signalled via the RhoA/Rho kinase pathway), increase cross-bridge cycling and development of force [55, 59]. Possible crosstalk between A_2a_ receptors and lidocaine may also involve transmembrane domains of adenyl cyclase and other downstream signalling pathways to alter intracellular free Ca^2+^ and/or myofibrillar sensitisation.

To our knowledge, little or no data exist on adenosine and lidocaine interactions in intact rat aortic rings or endothelial-vascular smooth muscle interactions. Adenosine A_2a_ and A_2b_ receptors are present on vascular endothelium and smooth muscle of many vessels [60, 61] and when activated can lead to vasodilation. A_2a_ receptor vasodilation is thought to involve: 1) endothelial NO production which activates smooth muscle guanylyl cyclase via opening Kir channels [61], and/or 2) more direct smooth muscle A_2a_ receptor activation which in turn stimulates mostly Gs proteins (and Gq) and cAMP signalling pathways to reduce intracellular Ca^2+^ levels [61, 62]. In addition, adenosine A_2a_ activation may activate sarcolemma Ca^2+^ channels and regulate influx in large elastic arteries and resistance vessels. Stella and colleagues showed that activation of A_2_ receptors stimulates protein kinase A to inhibit L-type Ca^2+^ channels in rod photoreceptors resulting in a decreased Ca^2+^ influx [63]. Gubitz and colleagues have proposed dual A_2a_ signalling involving the activation of both N- and P-type calcium channels by different G proteins and protein kinases in the some nerve terminals [64]. Gonvalves and colleagues showed that adenosine A_2a_ receptors facilitated Ca^2+^ uptake through class A calcium channels in rat hippocampal CA3 region [65].

Interestingly, Benkwitz and colleagues also showed that higher concentrations of lidocaine (1000 uM) in hamster oocytes potentiated Galpha_i_-coupled A_1_ receptor signalling by reducing cyclic AMP production in a dose-dependent manner through an unidentified mechanism [29]. The authors proposed that lidocaine was not an A1-receptor agonist *but enhanced adenosine-A1 receptor signalling*. They argued that lidocaine interacted with a pool of already activated Gαi present in the cytoplasm and thereby facilitated its ability to inhibit adenylate cyclase leading to lower cAMP [29]. We did not examine adenosine A1 receptor antagonism We conclude from our study that A_2a_ receptor may have enhanced lidocaine relaxation activation by directly effecting vascular smooth muscle (Fig. 6), and this may have occurred by reducing intracellular Ca^2+^ and/or myofibrillar contractile sensitization in intact isolated rat aortic rings, although the underlying mechanisms remain to be identified. Further studies are required to investigate the role of adenosine and lidocaine on membrane Ca^2+^ channel modulation in isolated rat aortic rings.

### Limitations of the study and possible physiological significance

The present study examined lidocaine relaxation in isolated rat thoracic rings using length-tension experiments and a number of antagonists of NO, prostanoids, K_v_, K_ATP_ and A_2_ receptors under normoxic and normal pH conditions from healthy rats. Before definitive conclusions can be drawn regarding the nature of unknown relaxation factor(s), it would be important to examine separately and in combination other drug antagonists and agonists of NO, prostanoids, K_v_, Sarc- and Mito-K_ATP_ channels and A_2_ receptors on lidocaine relaxation in intact and denuded rings. Furthermore, to gain greater mechanistic insight into the nature of voltage-dependent K^+^ channels and lidocaine vasorelaxation electrophysiological experiments would be essential. Leukotrienes, and leukotriene synthase inhibitors, may also be of interest because they have been shown to modulate rat aortic ring relaxation [66]. Possible physiological significance of the present study relates to lidocaine’s effect to regulate in vivo compliance such as ventricular-arterial coupling functions linking the heart as a pump to tissue perfusion [67, 68]. However, further in vivo studies are required to test this hypothesis. Also our work may have clinical applicability on the ancillary properties of lidocaine at the site of injection during infiltration, nerve block, or epidural anesthesia [14], and on damaged endothelium such as in plaque formation, arterial and venous conduit protection for cardiopulmonary bypass grafting [69], prevention of vascular spasm during neurosurgery [70], lowering elevated intracranial pressure [71], lidocaine cardioplegia [72, 73], and other surgical applications [54].

## Conclusions

We showed in isolated, oxygenated NE precontracted rat aortic rings that lidocaine relaxation was biphasic from 1 to 10 uM and 10 to 1000 uM. We further showed that lidocaine relaxation was significantly enhanced by endothelial removal, which did not appear to be NO or prostacyclin dependent. The putative unknown factor(s) responsible for enhanced relaxation may involve activation of smooth muscle voltage-sensitive K_v_ and 5-HD sensitive channels or pathways, and possible crosstalk with A_2a_ subtype receptor at higher lidocaine concentrations.

## Abbreviations

4-AP: 4-aminopyridine
5 HD: 5-Hydroxydecanoate
CSC: 8-(3-chlorostyryl) caffeine
MitoK_ATP_: Mitochondrial K_ATP_ channel
NE: Norepinephrine
NO: Nitric oxide
PSB-0788: 8-(4-(4-(4-chlorobenzyl)piperazine-1-sulfonyl)phenyl)-1-propylxanthine
SarcK_ATP_: Sarcoplasmic K_ATP_ channel
